# Supplementary material for: Genetically Engineered Probiotic Limosilactobacillus reuteri Releasing IL-22 (LR-IL-22) Modifies the Tumor Microenvironment, Enabling Irradiation in Ovarian Cancer
Source: Cancers (Basel). 2024 Jan 23;16(3):474. doi: 10.3390/cancers16030474 (PMC10854600; doi:10.3390/cancers16030474)
Supplement: Supplementary file 1 [file cancers-16-00474-s001.zip › cancers-2812287-supplementary.pdf]

Supplementary data:

Supplemental Table S1. Genes upregulated by TBI radiation in male mice that were specifically reversed/rescued by gavage of LR-IL-22. **(A) Non-mitochondrial genes. (B) Mitochondrial genes.**

(A) Non-mitochondrial genes

Gene Symbol	Name	Role
Rps19-ps6	Ribosomal protein S19, pseudogene 6	RPS19 protein is normally upregulated in human breast and ovarian cancer cells and released from apoptotic tumor cells where it interacts with the complement C5a receptor 1 expressed on tumor infiltrating myeloid-derived suppressor cells to promotes tumor growth by facilitating recruitment of these cells to tumors.<br>RPS19 induces the production of immunosuppressive cytokines, including TGF-β, by myeloid-derived suppressor cells in tumor-draining lymph nodes, leading to T cell responses skewed toward Th2 phenotypes.<br>RPS19 promotes generation of regulatory T cells while reducing infiltration of CD8+ T cells into tumors.<br>Conclusion: Reducing RPS19 in tumor cells or blocking the C5a receptor 1–RPS19 interaction will decrease RPS19-mediated immunosuppression, impair tumor growth, and delay the development of tumors.
Strip2	Striatin Interacting Protein 2	STRIP2 is a core component of the striatin-interacting phosphatase and kinase (STRIPAK) complexes, which is involved in tumor initiation and progression via the regulation of cell contractile and metastasis.<br>It motivates NSCLC progression by interacting with IGF2BP3 to regulate TMBIM6 mRNA stability in an m6A-dependent manner.
Folr1	Folate receptor alpha	Protein helps regulate transport of the B-vitamin folate into cells.<br>FOLR1 increases sensitivity to cisplatin treatment in ovarian cancer cells.<br>It is over-expressed in most ovarian cancers.

Tmem181c-ps	Transmembrane protein 181C, pseudogene	Mediates action of cytolethal distending toxins (CDT), which are secreted by many pathogenic bacteria. Expression level of TMEM181 is rate-limiting for intoxication.
S100a11	S100 calcium-binding protein A11	The protein encoded by this gene is a member of the S100 family of proteins containing 2 EF-hand calcium-binding motifs.<br>The protein may function in motility, invasion, and tubulin polymerization. Chromosomal rearrangements and altered expression of this gene have been implicated in tumor metastasis.<br>In most cancers, S100A11 is highly expressed and correlated to tumor promotion and progression. In Ovarian cancer, S100A11 was able to promote the growth, invasion and migration of ovarian cancer cells. Thus, knockdown of S100A11 expression suppresses ovarian cancer cell growth and invasion.
Gsta3	Glutathione S-transferase A3	GSTA3 inhibited hepatic stellate cells activation and liver fibrosis through suppression of the MAPK and GSK-3β signaling pathways.
Thnsl2	Threonine synthase like 2	Protein acts as a cytokine and can induce the production of the inflammatory cytokine IL6 in osteoblasts.
H2-Q6	Histocompatibility 2, Q region locus 6	Controls a lymph node and splenic lymphocyte antigen detected by BALB/cBy anti-ORA1-a tumor antibody.
Ptpn6	Tyrosine-protein phosphatase non-receptor type 6	Protein encoded by this gene is a member of the protein tyrosine phosphatase (PTP) family. PTPs are known to be signaling molecules that regulate a variety of cellular processes including cell growth, differentiation, mitotic cycle, and oncogenic transformation. This PTP is expressed primarily in hematopoietic cells, and functions as an important regulator of multiple signaling pathways in hematopoietic cells.<br>Protein associated with progression of esophageal squamous cell carcinoma.<br>PTPN6 is highly expressed in colorectal cancer tissues. High expression of PTPN6 is associated with poor prognosis in patients with colon cancer.
Pias4	Protein inhibitor of activated STAT 4	PIAS4 promotes tumorigenicity and metastasis of HCC.<br>PIAS4 is an activator of hypoxia signaling via VHL suppression during growth of pancreatic cancer cells.

Plk2	Polo-like kinase 2	In rats, PLK2 is highly induced in ovarian granulosa cells; overexpressed PLK2 blocks the cell cycle in the G0/G1 phase, while downregulation of it decreases the number of G0/G1 phase cells but increases the cell vitality.<br>In epithelial ovarian cancer, CpG island methylation caused PLK2 downregulation was related to paclitaxel and platinum tolerance and postoperative recurrence.<br>PLK2 expression was positively correlated to paclitaxel resistance resulting from its anti-proliferative effects during mitosis in ovarian cancer cell line A2780 and promoting tumor cell viability.
Msmo1	Methylsterol monooxygenase 1	Down-regulation of MSMO1 promotes the development and progression of pancreatic cancer.
Gngt2	G protein subunit gamma transducin 2	It acts as a tumor suppressor in breast cancer through stimulating MRAS signaling
Scand1	SCAN domain-containing protein 1	SCAND1 reverses epithelial-to-mesenchymal transition (EMT) and suppresses prostate cancer growth and migration.
Def8	Differentially Expressed In FDCP 8 Homolog	Involved in lysosome localization; positive regulation of bone resorption; and positive regulation of ruffle assembly
Rpl36a-ps3	Ribosomal protein L36A, pseudogene 3	Plays a regulatory role in the development of radioresistance in oral SCC.
Pid1	Phosphotyrosine Interaction Domain containing 1	Involved in several processes, including mitochondrion morphogenesis; negative regulation of phosphate metabolic process; and positive regulation of macromolecule metabolic process.<br>Growth-inhibitory gene in embryonal brain tumors and gliomas
Rac2	Ras-related C3 botulinum toxin substrate 2	Gene encodes a member of the Ras superfamily of small guanosine triphosphate (GTP)-metabolizing proteins.<br>RAC2 is upregulated in clear cell RCC tissues and cell lines, and elevated expression levels of RAC2 are associated with a poor overall survival, higher Tumor-Node-Metastasis stage and worse G grade in clear cell RCC.
Alox5ap	Arachidonate 5-lipoxygenase activating protein	Protein is necessary in synthesis of leukotriene, which are lipid mediators of inflammation that is involved in respiratory and cardiovascular diseases.

Map2k3	Mitogen-Activated Protein Kinase Kinase 3	The protein encoded by this gene is a dual specificity protein kinase that belongs to the MAP kinase kinase family. This kinase is activated by mitogenic and environmental stress, and participates in the MAP kinase-mediated signaling cascade. It phosphorylates and thus activates MAPK14/p38-MAPK. Expression of RAS oncogene is found to result in the accumulation of the active form of this kinase, which thus leads to the constitutive activation of MAPK14, and confers oncogenic transformation of primary cells.
Ddit4l	DNA-damage-inducible transcript 4 like	DDIT4L is a negative regulator of mTOR. It is a stress responsive protein whose expression is increased under the hypoxic condition and causes or sensitize towards cell death through the regulation mTOR activity and reduction of thioredoxin-1.<br>Ras-mediated transformation of human ovarian epithelial cells relied on DDIT4, which facilitated expression of autophagy-related proteins and inhibited tumor cell apoptosis.<br>The over-expression of DDIT4 in ovarian cancer was significantly related to serous cancer, advanced stage, ascites, partial or no response to chemotherapy, overall survival, and tumor-free survival.<br>DDIT4 is associated with cell invasion and migration of various human ovarian epithelial cancer cell lines.
St6galnac6	ST6 N-acetylgalactosaminide alpha-2,6-sialyltransferase 6	It belongs to a family of sialyltransferases that modify proteins and ceramides on the cell surface to alter cell-cell or cell-extracellular matrix interactions.
B3gnt7	β1-3-N-acetylglucosaminyltransferase 7	Predicted to enable UDP-glycosyltransferase activity.<br>Interleukin-22 regulates B3GNT7 expression to induce fucosylation of glycoproteins in intestinal epithelial cells.
Tacc1	Transforming Acidic Coiled-Coil Containing Protein 1	The function of this gene has not yet been determined; however, it is speculated that it may represent a breast cancer candidate gene. It is located close to FGFR1 on a region of chromosome 8 that is amplified in some breast cancers.
Gm9917	Predicted gene 9917	

Alox5	Arachidonate 5-Lipoxygenase	This gene encodes a member of the lipoxygenase gene family and plays a dual role in the synthesis of leukotrienes from arachidonic acid. The encoded protein catalyzes the conversion of arachidonic acid to 5(S)-hydroperoxy-6-trans-8,11,14-cis-eicosatetraenoic acid, and further to the allylic epoxide 5(S)-trans-7,9-trans-11,14-cis-eicosatetrenoic acid (leukotriene A4). Leukotrienes are important mediators of a number of inflammatory and allergic conditions.
Sh3bgrl3	SH3 Domain Binding Glutamate Rich Protein Like 3	Protein is up-regulated in GBM compared to normal cerebral tissue on proteomic analysis.<br>SH3BGRL3, transcribed by STAT3, facilitates glioblastoma tumorigenesis by activating STAT3 signaling.<br>SH3BGRPL3 protein as a post-translational modification of the 27kDa tumor necrosis factor alpha (TNF-α) inhibitory protein, TIP-B1. This protein is potentially involved in resistance of cells to the apoptosis-inducing effect of TNF-α.
Car13	Carbonic Anhydrase 13	Enables carbonate dehydratase activity. Predicted to be involved in one-carbon metabolic process. Located in cytosol; intracellular membrane-bounded organelle; and myelin sheath.
Gm7665	Predicted pseudogene 7665	
Krt23	Keratin 23	The protein encoded by this gene is a member of the keratin family and is responsible for the structural integrity of epithelial cells.<br>Knockdown of KRT23 renders colon cancer cells more sensitive to irradiation and reduces proliferation of the KRT23 depleted cells compared to irradiated control cells.
Rnf213	Ring finger protein 213	Encodes a protein containing a C3HC4-type RING finger domain, which is a specialized type of Zn-finger that binds two atoms of zinc and is thought to be involved in mediating protein-protein interactions.
Map3k6	Mitogen-Activated Protein Kinase Kinase Kinase 6	Encodes a serine/threonine protein kinase that forms a component of protein kinase-mediated signal transduction cascades. The encoded kinase participates in the regulation of vascular endothelial growth factor (VEGF) expression

Pgs1	Phosphatidylglycerophosphate synthase 1	Predicted to enable CDP-diacylglycerol-glycerol-3-phosphate 3-phosphatidyltransferase activity and calcium ion binding activity. Predicted to be involved in cardiolipin biosynthetic process and diacylglycerol metabolic process. Located in endoplasmic reticulum.
Clca3a1	Chloride channel accessory 3A1	This protein is not expressed in humans but is in certain other species such as mouse.
Reep5	Receptor Accessory Protein 5	Involved in endoplasmic reticulum organization and regulation of intracellular transport.
Mdm2	Mouse double minute 2 homolog	Negative regulator of the p53 tumor suppressor.
Vmn2r26	Vomeronasal 2, receptor 26	Intestinal Tuft-2 Cells Exert Antimicrobial Immunity Via Sensing Bacterial Metabolite N-Undecanoylglycine by Vomeronasal Receptor Vmn2r26
S100a13	S100 calcium binding protein A13	Up-regulation of S100A13 was detected in cystic papillary thyroid carcinoma[9] and association of S100A13 expression and chemotherapy resistance was shown in proteomics study of melanoma.<br>High mRNA expression of S100A1, S100A3, S100A5, S100A6, and S100A13 were significantly correlated with better overall survival in ovarian cancer.
Ogt	O-Linked N-Acetylglucosamine (GlcNAc) Transferase	Encodes a glycosyltransferase that catalyzes the addition of a single N-acetylglucosamine in O-glycosidic linkage to serine or threonine residues.<br>has been shown to exaggerate inflammatory response by counteracting anti-inflammatory signaling such as STAT3 (signal transducer and activator of transcription 3) signaling in innate immune cells.
Mfsd2a	Major facilitator superfamily domain-containing protein 2	Membrane transport protein that is expressed in the endothelium of the blood–brain barrier (BBB) and has an essential role in BBB formation and function.
Agt	Angiotensinogen	The protein encoded by this gene, pre-angiotensinogen or angiotensinogen precursor, is expressed in the liver and is cleaved by the enzyme renin in response to lowered blood pressure.

Reg3g	Regenerating islet-derived protein 3 gamma	Intestinal paneth cells produce REG3G (or REG3 gamma) via stimulation of toll-like receptors (TLRs) by pathogen-associated molecular patterns (PAMPs). REG3 gamma specifically targets Gram-positive bacteria because it binds to their surface peptidoglycan layer. Bacteria engineered to produce IL-22 in intestine induce expression of REG3G to reduce ethanol-induced liver disease in mice.
Gm44867		
Tmem229a	Transmembrane Protein 229A	Predicted to be integral component of membrane.<br>TMEM229A suppresses non-small cell lung cancer progression via inactivating the ERK pathway
Dync1li2	Dynein cytoplasmic 1 light intermediate chain 2	Cytoplasmic dynein is a microtubule-associated motor protein.
Atg9b	Autophagy Related 9B	This gene functions in the regulation of autophagy, a lysosomal degradation pathway. This gene also functions as an antisense transcript in the posttranscriptional regulation of the endothelial nitric oxide synthase 3 gene, which has 3' overlap with this gene on the opposite strand. Mutations in this gene and disruption of the autophagy process have been associated with multiple cancers.
Thoc5	THO complex 5	A member of THO complex which is a subcomplex of the transcription/export complex (TREX)
Irgm2	Immunity Related GTPase M	This gene encodes a member of the p47 immunity-related GTPase family. The encoded protein may play a role in the innate immune response by regulating autophagy formation in response to intracellular pathogens.
Ttc39a	Tetratricopeptide Repeat Domain 39A	The function of TTC39A is currently not well understood. The main feature within tetratricopeptide repeat 39A is the domain of unknown function 3808 (DUF3808), spanning almost the entire protein.
Rab27a	Ras-Related Protein Rab-27A	The protein encoded by this gene belongs to the small GTPase superfamily, Rab family. The protein is membrane-bound and may be involved in protein transport and small GTPase mediated signal transduction.

Phip	Pleckstrin Homology Domain Interacting Protein	Encodes two protein-isoforms, PHIP/DCAF14 and NDRP, each involved in neurodevelopmental processes, including E3 ubiquitination and neuronal differentiation.
Lect2	Leukocyte cell-derived chemotaxin 2	This gene encodes a secreted, 16 kDa protein that acts as a chemotactic factor to neutrophils and stimulates the growth of chondrocytes and osteoblasts.
Limd2	LIM Domain Containing 2	LIMD2 is involved in the signal transduction cascade that links integrin-mediated signaling to cell motility/metastatic behavior.
Ackr4	Atypical Chemokine Receptor 4	ACKR4 restrains antitumor immunity by regulating CCL21
Eppk1	Epiplakin 1	Role in the organization of cytoskeletal architecture.<br>Epiplakin has been found to bind to keratin filaments and may contribute to inhibiting the growth of the filaments. Blocking the expression of epiplakin in cultured corneal epithelial cells via siRNA has been associated with faster wound closure and faster migration of corneal cells.
Tubb6	Tubulin Beta 6 Class V	Predicted to enable GTP binding activity. Predicted to be a structural constituent of cytoskeleton. Predicted to be involved in microtubule cytoskeleton organization and mitotic cell cycle. Located in microtubule.
Fam118b	Family With Sequence Similarity 118 Member B	Enables identical protein binding activity. Predicted to be involved in Cajal body organization. Predicted to be located in Cajal body.
C2	Complement C2	The protein encoded by this gene is part of the classical pathway of the complement system, acting as a multi-domain serine protease.
Izumo4	IZUMO Family Member 4	Located in nucleus.
Casp4	Caspase 4	This gene encodes a protein that is a member of the cysteine-aspartic acid protease (caspase) family. Sequential activation of caspases plays a central role in the execution-phase of cell apoptosis.<br>It displays increased intestinal expression during IBD and have been implicated in chronic IBD inflammation.
Atxn2l	Ataxin 2 Like	This gene encodes an ataxin type 2 related protein of unknown function. This protein is a member of the spinocerebellar ataxia (SCAs) family, which is associated with a complex group of neurodegenerative disorders.

Snrnp25	Small Nuclear Ribonucleoprotein U11/U12 Subunit 25	Protein coding gene.
Rbck1	RANBP2-Type And C3HC4-Type Zinc Finger Containing 1	RBCK1 is important for the ubiquitination of PXR and may play a role in its proteasomal degradation.
Oas2	2'-5'-Oligoadenylate Synthetase 2	This gene encodes a member of the 2-5A synthetase family, essential proteins involved in the innate immune response to viral infection.
Arl5c	ADP Ribosylation Factor Like GTPase 5C	Predicted to enable GTP binding activity. Predicted to be involved in intracellular protein transport; protein localization to Golgi membrane; and vesicle-mediated transport. Predicted to be active in trans-Golgi network.
Gmip	GEM Interacting Protein	This gene encodes a member of the ARHGAP family of Rho/Rac/Cdc42-like GTPase activating proteins. The encoded protein interacts with the Ras-related protein Gem through its N-terminal domain. Separately, it interacts with RhoA through a RhoGAP domain, and stimulates RhoA-dependent GTPase activity.
Neurl1a	Neuralized E3 Ubiquitin Protein Ligase 1	Neuralized family member NEURL1 is a ubiquitin ligase for the cGMP-specific phosphodiesterase 9A
Tmem158	Transmembrane Protein 158	Constitutive activation of the Ras pathway triggers an irreversible proliferation arrest reminiscent of replicative senescence. Transcription of this gene is upregulated in response to activation of the Ras pathway, but not under other conditions that induce senescence.
Vav1	Vav Guanine Nucleotide Exchange Factor 1	Is a key signal transducer downstream of the T cell antigen receptor
Kctd12	Potassium Channel Tetramerization Domain Containing 12	KCTD12 promotes tumorigenesis by facilitating CDC25B/CDK1/Aurora A-dependent G2/M transition. Aurora A interacts with BRCA1 to induce breast tumorigenesis37 and regulates the transcription of human telomerase reverse transcriptase through c-myc pathways in ovarian and breast epithelial carcinomas.
Acta1	Actin Alpha 1, Skeletal Muscle	The product encoded by this gene belongs to the actin family of proteins, which are highly conserved proteins that play a role in cell motility, structure and integrity.
Sytl3	Synaptotagmin Like 3	The protein encoded by this gene belongs to a family of peripheral membrane proteins that play a role in vesicular trafficking.

Spryd3	SPRY domain-containing protein 3	Predicted to be involved in cell surface receptor signaling pathway and cytoskeleton organization. Predicted to be active in cytoplasm.
Ppip5k1	Diphosphoinositol Pentakisphosphate Kinase 1	This gene encodes a dual functional inositol kinase. The encoded enzyme converts inositol hexakisphosphate to diphosphoinositol pentakisphosphate and diphosphoinositol pentakisphosphate to bis-diphosphoinositol tetrakisphosphate. This protein may be important for intracellular signaling pathways.
Apol9b	Apolipoprotein L9b	Typically associated with the transport of lipids in the organism.
Ctso	Cathepsin O	A proteolytic enzyme involved in cellular protein degradation and turnover.
Nudt7	Nudix hydrolase 7	Nudix hydrolases eliminate potentially toxic nucleotide metabolites from the cell and regulate the concentrations and availability of many different nucleotide substrates, cofactors, and signaling molecules.
Sct	Secretin	This gene encodes a member of the glucagon family of peptides. The encoded preproprotein is secreted by endocrine S cells in the proximal small intestinal mucosa as a prohormone, then proteolytically processed to generate the mature peptide hormone. The release of this active peptide hormone is stimulated by either fatty acids or acidic pH in the duodenum. This hormone stimulates the secretion of bile and bicarbonate in the duodenum, pancreatic and biliary ducts.
Dvl1	Dishevelled segment polarity protein 1	Encodes a cytoplasmic phosphoprotein that regulates cell proliferation, acting as a transducer molecule for developmental processes, including segmentation and neuroblast specification.
Krt8-ps	Keratin 8	This gene is a member of the type II keratin family clustered on the long arm of chromosome 12. Type I and type II keratins heteropolymerize to form intermediate-sized filaments in the cytoplasm of epithelial cells. The product of this gene typically dimerizes with keratin 18 to form an intermediate filament in simple single-layered epithelial cells. This protein plays a role in maintaining cellular structural integrity and also functions in signal transduction and cellular differentiation.
Ccng1	Cyclin G1	CCNG1 overexpression, which is associated with poor clinical prognosis of high-grade serous ovarian cancer, can promote metastasis and chemotherapy resistance via the P53mt-Notch3 pathway.

Hgfac	HGF Activator	This gene encodes a member of the peptidase S1 protein family. The encoded protein is first synthesized as an inactive single-chain precursor before being activated to a heterodimeric form by endoproteolytic processing. It acts as serine protease that converts hepatocyte growth factor to the active form.
S100a6	S100 calcium-binding protein A6	The protein encoded by this gene is a member of the S100 family of proteins containing 2 EF-hand calcium-binding motifs. S100 proteins are localized in the cytoplasm and/or nucleus of a wide range of cells, and involved in the regulation of a number of cellular processes such as cell cycle progression and differentiation.
Cacna1a	Calcium Voltage-Gated Channel Subunit Alpha1 A	Voltage-dependent calcium channels mediate the entry of calcium ions into excitable cells, and are also involved in a variety of calcium-dependent processes, including muscle contraction, hormone or neurotransmitter release, and gene expression.
Lamtor4	Late Endosomal/Lysosomal Adaptor, MAPK And MTOR Activator 4	Contributes to guanyl-nucleotide exchange factor activity and molecular adaptor activity. Involved in several processes, including cellular response to amino acid stimulus; positive regulation of TOR signaling; and protein localization to lysosome. Located in lysosome.
Myo1b	Myosin IB	Enables ATP binding activity; actin filament binding activity; and microfilament motor activity. Involved in actin filament organization and post-Golgi vesicle-mediated transport. Located in several cellular components, including actin filament; endosome; and perinuclear region of cytoplasm.
Akr1b7	Aldo-keto reductase family 1, member B7	Predicted to enable several functions, including alcohol dehydrogenase (NADP+) activity; geranylgeranyl reductase activity; and indanol dehydrogenase activity. Predicted to be involved in several processes, including cellular detoxification of aldehyde; daunorubicin metabolic process; and primary alcohol metabolic process.<br>Nuclear receptor PXR targets AKR1B7 to protect mitochondrial metabolism and renal function in AKI.
Rtp4	Receptor Transporter Protein 4	Predicted to enable olfactory receptor binding activity. Involved in defense response to virus; detection of chemical stimulus involved in sensory perception of bitter taste; and protein targeting to membrane.

Ccdc28b	Coiled-Coil Domain Containing 28B	The product of this gene localizes to centrosomes and basal bodies. The protein colocalizes with several proteins associated with Bardet-Biedl syndrome (BBS) and participates in the regulation of cilia development.
Gsta4	Glutathione S-Transferase Alpha 4	This gene encodes a glutathione S-tranferase belonging to the alpha class. The alpha class genes, which are located in a cluster on chromosome 6, are highly related and encode enzymes with glutathione peroxidase activity that function in the detoxification of lipid peroxidation products.
Ido1	Indoleamine 2,3-Dioxygenase 1	Encodes a heme-containing enzyme physiologically expressed in a number of tissues and cells, such as the small intestine, lungs, female genital tract or placenta.<br>Indoleamine 2,3-dioxygenase is the first and rate-limiting enzyme of tryptophan catabolism through the kynurenine pathway.
Fbxw9	F-Box And WD Repeat Domain Containing 9	Silencing of FBXW9 inhibited cell proliferation and cell cycle progression in breast cancer cells.
Ano7	Anoctamin 7	This prostate-specific gene encodes a cytoplasmic protein, as well as a polytopic membrane protein which may serve as a target in prostate cancer diagnosis and immunotherapy.
S100a1	S100 Calcium Binding Protein A1	S100A1 is a member of the S100 family of calcium-binding proteins.
Dynll1	Dynein Light Chain LC8-Type 1	Cytoplasmic dyneins are large enzyme complexes with a molecular mass of about 1,200 kD. They contain two force-producing heads formed primarily from dynein heavy chains, and stalks linking the heads to a basal domain, which contains a varying number of accessory intermediate chains. The protein described in this record is a light chain and exists as part of this complex but also physically interacts with and inhibits the activity of neuronal nitric oxide synthase.
Ftl1-ps2	Ferritin light polypeptide, pseudogene 2	Coded for a protein that plays a central role in iron uptake and storage
Gm42047	Predicted gene, 42047	
Kank3	KN motif and ankyrin repeat domains 3	Predicted to be involved in negative regulation of actin filament polymerization.

Rhog	Ras Homolog Family Member G	This gene encodes a member of the Rho family of small GTPases, which cycle between inactive GDP-bound and active GTP-bound states and function as molecular switches in signal transduction cascades. Rho proteins promote reorganization of the actin cytoskeleton and regulate cell shape, attachment, and motility.
Rps26-ps1	Ribosomal protein S26, pseudogene 1	Codes for a ribosomal subunit structural protein involved in the growth and development process.
Gm8399	Predicted gene 8399	
Arhgap27	Rho GTPase activating protein 27	This gene encodes a member of a large family of proteins that activate Rho-type guanosine triphosphate (GTP) metabolizing enzymes. The encoded protein may pay a role in clathrin-mediated endocytosis.
Abhd4	Abhydrolase Domain Containing 4, N-Acyl Phospholipase B	Predicted to enable lysophosphatidic acid acyltransferase activity and lysophospholipase activity.
Trim38	Tripartite Motif Containing 38	The encoded protein contains a RING-type zinc finger, B box-type zinc finger and SPRY domain. The function of this protein has not been identified.
Hivep3	HIVEP Zinc Finger 3	This gene encodes a member of the human immunodeficiency virus type 1 enhancer-binding protein family. Members of this protein family contain multiple zinc finger and acid-rich (ZAS) domains and serine-threonine rich regions. This protein acts as a transcription factor and is able to regulate nuclear factor kappaB-mediated transcription by binding the kappaB motif in target genes. This protein also binds the recombination signal sequence that flanks the V, D, and J regions of immunoglobulin and T-cell receptors.
Gm17847	Predicted gene, 17847	
Tjap1	Tight junction associated protein 1	This gene encodes a tight junction-associated protein. Incorporation of the encoded protein into tight junctions occurs at a late stage of formation of the junctions. The encoded protein localizes to the Golgi and may function in vesicle trafficking.
Cpvl	Carboxypeptidase Vitellogenic Like	The protein encoded by this gene is a carboxypeptidase and bears strong sequence similarity to serine carboxypeptidases. Carboxypeptidases are a large class of proteases that act to cleave a single amino acid from the

		carboxy termini of proteins or peptides. The exact function of this protein, however, has not been determined.
Dgkq	Diacylglycerol Kinase Theta	The protein encoded by this gene contains three cysteine-rich domains, a proline-rich region, and a pleckstrin homology domain with an overlapping Ras-associating domain. It is localized in the speckle domains of the nucleus, and mediates the regeneration of phosphatidylinositol (PI) from diacylglycerol in the PI-cycle during cell signal transduction.
Pou2f3	POU Class 2 Homeobox 3	This gene encodes a member of the POU domain family of transcription factors. POU domain transcription factors bind to a specific octamer DNA motif and regulate cell type-specific differentiation pathways. The encoded protein is primarily expressed in the epidermis, and plays a critical role in keratinocyte proliferation and differentiation.
Zfhx3	Zinc Finger Homeobox 3	This gene encodes a transcription factor with multiple homeodomains and zinc finger motifs, and regulates myogenic and neuronal differentiation.
Gprc5c	G Protein-Coupled Receptor Class C Group 5 Member C	The protein encoded by this gene is a member of the type 3 G protein-coupled receptor family. Members of this superfamily are characterized by a signature 7-transmembrane domain motif. The specific function of this protein is unknown.
Ccdc129	Coiled-coil domain containing 129	Protein involved in intercellular transmembrane signal transduction and genetic signal transcription, among other functions. Alterations in expression, mutation, and DNA promoter methylation of CCDC family genes have been shown to be associated with the pathogenesis of many diseases, including primary ciliary dyskinesia, infertility, and tumors. In recent studies, CCDC family genes have been found to be involved in regulation of growth, invasion, metastasis, chemosensitivity, and other biological behaviors of malignant tumor cells in various cancer types, including nasopharyngeal carcinoma, lung cancer, colorectal cancer, and thyroid cancer.

Krt18	Keratin 18	KRT18 encodes the type I intermediate filament chain keratin 18. Keratin 18, together with its filament partner keratin 8, are perhaps the most commonly found members of the intermediate filament gene family. They are expressed in single layer epithelial tissues of the body.
Gm13657	Predicted gene 13657	
Gm15590	Predicted gene 15590	
Kdm5b	Lysine Demethylase 5B	This gene encodes a lysine-specific histone demethylase that belongs to the jumonji/ARID domain-containing family of histone demethylases. The encoded protein is capable of demethylating tri-, di- and monomethylated lysine 4 of histone H3. This protein plays a role in the transcriptional repression or certain tumor suppressor genes and is upregulated in certain cancer cells.
Zfp281	Zinc finger protein 281	Enables DNA-binding transcription repressor activity, RNA polymerase II-specific and RNA polymerase II cis-regulatory region sequence-specific DNA binding activity.
Pold4	DNA Polymerase Delta 4, Accessory Subunit	It is a component of the DNA polymerase delta complex.
Fam160a1	FHF Complex Subunit HOOK Interacting Protein 1A	FHF Complex Subunit HOOK Interacting Protein 1A
Hmx3	H6 Family Homeobox 3	Predicted to enable DNA-binding transcription factor activity, RNA polymerase II-specific and RNA polymerase II transcription regulatory region sequence-specific DNA binding activity. Predicted to be involved in ear development and regulation of transcription by RNA polymerase II.
Gm10382	Predicted gene 10382	
Pfkfb3	6-Phosphofructo-2-Kinase/Fructose-2,6-Biphosphatase 3	PFKFB3 blockade inhibits hepatocellular carcinoma growth by impairing DNA repair through AKT
Reg3b	Regenerating islet-derived 3 beta	Involved in defense response to Gram-negative bacterium and defense response to Gram-positive bacterium.
Bicd1	BICD cargo adaptor 1	This gene encodes an adaptor protein that belongs to the bicaudal D family of dynein cargo adaptors. The encoded protein acts as an intracellular cargo transport cofactor that regulates the microtubule-based

		loading of cargo onto the dynein motor complex. It also controls dynein motor activity and coordination.
Clmn	Calmin	Protein Coding gene. Diseases associated with CLMN include Psoriasis 2 and Psoriasis 3.
Bax	Bcl-2 Associated X-protein	A pro-apoptotic member of the Bcl-2 gene family; it encodes a 21-kDa protein named BAX-alpha, whose association with Bcl-2 researchers believe plays a critical role in regulating intrinsic apoptosis.
Lypd8	Ly6/PLAUR domain containing 8	Lypd8 mediates segregation of intestinal bacteria and epithelial cells in the colon to preserve intestinal homeostasis.
Adgrg2	Adhesion G Protein-Coupled Receptor G2	This gene encodes a member of the G protein-coupled receptor family described as an epididymis-specific transmembrane protein. The encoded protein may be proteolytically processed as it contains a motif shown to be a protein scission motif in some members of this family.
Arhgef28	Rho guanine nucleotide exchange factor 28	This gene encodes a member of the Rho guanine nucleotide exchange factor family. The encoded protein interacts with low molecular weight neurofilament mRNA and may be involved in the formation of amyotrophic lateral sclerosis neurofilament aggregates.
Ascc3	Activating Signal Cointegrator 1 Complex Subunit 3	This gene encodes a protein that belongs to a family of helicases that are involved in the ATP-dependent unwinding of nucleic acid duplexes. The encoded protein is the largest subunit of the activating signal cointegrator 1 complex that is involved in DNA repair and resistance to alkylation damage.
Plekhg1	Pleckstrin Homology And RhoGEF Domain Containing G1	Predicted to enable guanyl-nucleotide exchange factor activity. Predicted to be involved in regulation of small GTPase mediated signal transduction. Located in nucleoplasm.

(B) Mitochondrial genes

Symbol	Name	Role
Mcub	Mitochondrial Calcium Uniporter Dominant Negative Subunit Beta	Predicted to enable calcium channel inhibitor activity. Predicted to be involved in calcium import into the mitochondrion and mitochondrial calcium ion homeostasis. Located in mitochondrion and nucleoplasm. Is integral component of mitochondrial inner membrane. Part of uniplex complex. It regulates the molecular composition of the mitochondrial calcium uniporter channel to limit mitochondrial calcium overload during stress.
Ogdhl	Oxoglutarate Dehydrogenase L	The protein encoded by this gene is similar to oxoglutarate dehydrogenase (OGDH) of the OGDH complex, which degrades glucose and glutamate. This gene encodes several isoforms, including some that appear to localize to mitochondria. The encoded protein down-regulates the AKT signaling cascade and can suppress the growth of cervical cancer cells.
Pklr	Pyruvate Kinase L/R	The protein encoded by this gene is a pyruvate kinase that catalyzes the transphosphorylation of phohsphoenolpyruvate into pyruvate and ATP, which is the rate-limiting step of glycolysis.
Akr1b7	Aldo-keto reductase family 1, member B7	Predicted to enable several functions, including alcohol dehydrogenase (NADP+) activity; geranylgeranyl reductase activity; and indanol dehydrogenase activity. Predicted to be involved in several processes, including cellular detoxification of aldehyde; daunorubicin metabolic process; and primary alcohol metabolic process.<br>Nuclear receptor PXR targets AKR1B7 to protect mitochondrial metabolism and renal function in AKI.

Supplemental Table S2. Genes downregulated by TBI radiation in male mice that were specifically reversed/rescued by gavage of LR-IL-22. **(A) Non-mitochondrial genes. (B) Mitochondrial genes.**

(A) Non-mitochondrial genes

Gene Symbol	Name	Role
Pde5a	PDE5A phosphodiesterase 5A	This gene encodes a cGMP-binding, cGMP-specific phosphodiesterase, a member of the cyclic nucleotide phosphodiesterase family. This phosphodiesterase specifically hydrolyzes cGMP to 5'-GMP. It is involved in the regulation of intracellular concentrations of cyclic nucleotides and is important for smooth muscle relaxation in the cardiovascular system.
Vps13b	Vacuolar protein sorting 13 homolog B	This gene encodes a potential transmembrane protein that may function in vesicle-mediated transport and sorting of proteins within the cell. This protein may play a role in the development and the function of the eye, hematological system, and central nervous system.
Xrn1	5'-3' Exoribonuclease 1	This gene encodes a member of the 5'-3' exonuclease family. The encoded protein may be involved in replication-dependent histone mRNA degradation, and interacts directly with the enhancer of mRNA-decapping protein 4.
Tmem252	Transmembrane Protein 252	Integral component of membrane and is expressed mainly in the kidney and small intestine of humans.<br>TMEM252 was found to be one of six novel genes associated with the prognosis of triple-negative breast cancer.

Tsc22d1	TSC22 Domain Family Member 1	Predicted to enable DNA-binding transcription activator activity, RNA polymerase II-specific and RNA polymerase II cis-regulatory region sequence-specific DNA binding activity.
Slc27a2	Solute Carrier Family 27 Member 2	The protein encoded by this gene is an isozyme of long-chain fatty-acid-coenzyme A ligase family. It is expressed primarily in liver and kidney, and is present in both endoplasmic reticulum and peroxisomes, but not in mitochondria.
Mapk8	Mitogen-activated protein kinase 8	The protein encoded by this gene is a member of the MAP kinase family. MAP kinases act as an integration point for multiple biochemical signals, and are involved in a wide variety of cellular processes such as proliferation, differentiation, transcription regulation and development.
Epc2	Enhancer of polycomb homolog 2	EPC2 is a component of a complex that directly or indirectly serves to prevent MYC protein accumulation and AML cell apoptosis, thus sustaining oncogenic potential.
Ptpn21	Protein Tyrosine Phosphatase Non-Receptor Type 21	The protein encoded by this gene is a member of the protein tyrosine phosphatase (PTP) family. PTPs are known to be signaling molecules that regulate a variety of cellular processes including cell growth, differentiation, mitotic cycle, and oncogenic transformation.
Tra2a	Transformer 2 Alpha Homolog	This gene is a member of the transformer 2 homolog family and encodes a protein with several RRM (RNA recognition motif) domains. This phosphorylated nuclear protein binds to specific RNA sequences and plays a role in the regulation of pre-mRNA splicing.

Usp42	Ubiquitin Specific Peptidase 42	Enables thiol-dependent deubiquitinase. Involved in protein deubiquitination. Predicted to be located in nucleoplasm.
Saysd1	SAYSVFN Motif Domain Containing 1	Located in intracellular membrane-bounded organelle. SAYSD1 is predominantly expressed in spermatids but is dispensable for fertility in mice
Slc31a1	Solute Carrier Family 31 Member 1	The protein encoded by this gene is a high-affinity copper transporter found in the cell membrane. The encoded protein functions as a homotrimer to effect the uptake of dietary copper.
Smpd2	Sphingomyelin Phosphodiesterase 2	Biological function is less likely to be as a sphingomyelinase and instead as a lysophospholipase
Ostm1	Osteoclastogenesis Associated Transmembrane Protein 1	This gene encodes a protein that may be involved in the degradation of G proteins via the ubiquitin-dependent proteasome pathway. The encoded protein binds to members of subfamily A of the regulator of the G-protein signaling (RGS) family through an N-terminal leucine-rich region.
Scrn3	Secernin-3	SCRN3 belongs to the peptidase C69 family and the secernin subfamily.[5] As a part of this family, the protein is predicted to enable cysteine-type exopeptidase activity and dipeptidase activity, as well as be involved in proteolysis.
Dyrk1a	Dual specificity tyrosine-phosphorylation-regulated kinase 1A	DYRK1A is localized in the Down syndrome critical region of chromosome 21, and is considered to be a strong candidate gene for learning defects associated with Down syndrome.
Nr2c2	Nuclear Receptor Subfamily 2 Group C Member 2	This gene encodes a protein that belongs to the nuclear hormone receptor family. Members of this family act as ligand-activated transcription factors and function in many

		biological processes such as development, cellular differentiation and homeostasis. The activated receptor/ligand complex is translocated to the nucleus where it binds to hormone response elements of target genes.<br>The protein encoded by this gene plays a role in protecting cells from oxidative stress and damage induced by ionizing radiation.
Ift46	Intraflagellar Transport 46	IFT46 plays an essential role in cilia development
Socs4	Suppressor Of Cytokine Signaling 4	The protein encoded by this gene contains a SH2 domain and a SOCS BOX domain. The protein thus belongs to the suppressor of cytokine signaling (SOCS), also known as STAT-induced STAT inhibitor (SSI), protein family. SOCS family members are known to be cytokine-inducible negative regulators of cytokine signaling.
Plce1	Phospholipase C Epsilon 1	PLCE1 regulates the migration, proliferation, and differentiation of podocytes
Wrn	WRN RecQ Like Helicase	The encoded nuclear protein is important in the maintenance of genome stability and plays a role in DNA repair, replication, transcription and telomere maintenance.
Dgcr6	DiGeorge syndrome critical region gene 6	The product of this gene shares homology with the Drosophila melanogaster gonadal protein, which participates in gonadal and germ cell development, and with the gamma-1 subunit of human laminin. This gene is a candidate for involvement in DiGeorge syndrome pathology and in schizophrenia.

Zbtb40	Zinc Finger And BTB Domain Containing 40	Predicted to enable DNA-binding transcription factor activity, RNA polymerase II-specific and RNA polymerase II cis-regulatory region sequence-specific DNA binding activity. Involved in cellular response to DNA damage stimulus. Located in nucleus.
Snph	Syntaphilin	Syntaxin-1, synaptobrevin/VAMP, and SNAP25 interact to form the SNARE complex, which is required for synaptic vesicle docking and fusion. The protein encoded by this gene is membrane-associated and inhibits SNARE complex formation by binding free syntaxin-1. Expression of this gene appears to be brain-specific.
Pcmtd2	Protein-L-Isoaspartate (D-Aspartate) O-Methyltransferase Domain Containing 2	Predicted to enable protein-L-isoaspartate (D-aspartate) O-methyltransferase activity. Predicted to be involved in protein methylation.
1700020I14Rik	Long intergenic noncoding RNAs (lincRNAs) 1700020I14Rik	
Dnajc4	DnaJ Heat Shock Protein Family (Hsp40) Member C4	Predicted to be involved in response to unfolded protein. Predicted to be integral component of membrane.
Nat9	N-Acetyltransferase 9	Involved in protein acetylation.
Plekhg2	Pleckstrin Homology And RhoGEF Domain Containing G2	The protein encoded by this gene is a RhoGTPase that can activate CDC42 by promoting exchange of GDP for GTP on CDC42. The encoded protein is activated by binding to the beta and gamma subunits of heterotrimeric guanine nucleotide-binding protein. Defects in this gene have been associated with leukodystrophy and acquired microcephaly with or without dystonia.

D17H6S53E	Chromosome 6 open reading frame 47	Ubiquitous expression in duodenum adult
Samd8	Sterile Alpha Motif Domain Containing 8	SAMD8 is an endoplasmic reticulum (ER) transferase that has no sphingomyelin synthase activity but can convert phosphatidylethanolamine (PE) and ceramide to ceramide phosphoethanolamine (CPE) albeit with low product yield. Appears to operate as a ceramide sensor to control ceramide homeostasis in the endoplasmic reticulum rather than a converter of ceramides
Crebrf	CREB3 Regulatory Factor	Enables DNA-binding transcription activator activity, RNA polymerase II-specific and RNA polymerase II transcription regulatory region sequence-specific DNA binding activity. Acts as a negative regulator of the endoplasmic reticulum stress response or unfolded protein response (UPR).
Taf15	TATA-Box Binding Protein Associated Factor 15	This gene encodes a member of the TET family of RNA-binding proteins. The encoded protein plays a role in RNA polymerase II gene transcription as a component of a distinct subset of multi-subunit transcription initiation factor TFIID complexes. Translocations involving this gene play a role in acute leukemia and extraskeletal myxoid chondrosarcoma, and mutations in this gene may play a role in amyotrophic lateral sclerosis.
Blvrb	Biliverdin Reductase B	Enables biliverdin reductase (NAD(P)+) activity and riboflavin reductase (NADPH) activity. Involved in heme catabolic process. Located in cytosol; nucleoplasm; and plasma membrane.
N4bp2l1	NEDD4 Binding Protein 2 Like 1	N4BP2L1 interacts with dynactin and contributes to GLUT4 trafficking and glucose uptake in adipocytes

(B) Mitochondrial genes

Symbol	Name	Role
Coa6	Cytochrome C Oxidase Assembly Factor 6	This gene encodes a member of the cytochrome c oxidase subunit 6B family. The encoded protein associates with cytochrome c oxidase may act has an cytochrome c oxidase mitochondrial respiratory complex VI assembly factor.
Pisd	Phosphatidylserine Decarboxylase	The protein encoded by this gene catalyzes the conversion of phosphatidylserine to phosphatidylethanolamine in the inner mitochondrial membrane. The encoded protein is active in phospholipid metabolism and interorganelle trafficking of phosphatidylserine.<br>Disruption of the Phosphatidylserine Decarboxylase Gene in Mice Causes Embryonic Lethality and Mitochondrial Defects.
Cyp2u1	Cytochrome P450, family 2, subfamily U, polypeptide 1	This gene encodes a member of the cytochrome P450 superfamily of enzymes.<br>CYP2U1 metabolized arachidonic acid, docosahexaenoic acid (DHA), and other long chain fatty acids which suggests that CYP2U1 may play a role in brain and immune functions.
Acsf2	Acyl-CoA Synthetase Family Member 2	Enables medium-chain fatty acid-CoA ligase activity. Predicted to be involved in fatty acid metabolic process. Predicted to be located in mitochondrial matrix.

Supplemental Table S3: GO (Gene Ontology) pathways associated with down regulated genes rescued by LR-IL-22 in irradiated GFP+ stem cells:

	GO.ID	Term	Annotated	Significant	Expected	raw.p.value
	GO:0010976	positive regulation of neuron projection development	5	5	5.0	0.0037
	GO:0010838	positive regulation of keratinocyte proliferation	1	1	1.0	0.0057
	GO:0045617	negative regulation of keratinocyte differentiation	1	1	1.0	0.0057
*	GO:0090303	positive regulation of wound healing	1	1	1.0	0.0057
	GO:0002755	MyD88-dependent toll-like receptor signaling pathway	2	2	2.0	0.0057
	GO:0051838	cytolysis by host of symbiont cells	2	2	2.0	0.0068
*	GO:0006953	acute-phase response	2	2	2.0	0.0068
	GO:0061844	antimicrobial humoral immune response mediated by antimicrobial peptide	2	2	2.0	0.0068
*	GO:0044278	cell wall disruption in another organism	2	2	2.0	0.0068
	GO:0050830	defense response to Gram-positive bacterium	4	4	4.0	0.0072
*	GO:0007584	response to nutrient	3	3	3.0	0.0100
	GO:0031115	negative regulation of microtubule polymerization	1	1	1.0	0.0114
	GO:0044805	late nucleophagy	1	1	1.0	0.0170
	GO:0034497	protein localization to phagophore assembly site	1	1	1.0	0.0170
	GO:0000422	autophagy of mitochondrion	2	2	2.0	0.0171
	GO:0006869	lipid transport	6	6	6.0	0.0175
	GO:0006629	lipid metabolic process	24	24	24.0	0.0234
	GO:0016525	negative regulation of angiogenesis	3	3	3.0	0.0248
	GO:0045742	positive regulation of epidermal growth factor receptor signaling pathway	3	3	3.0	0.0267
	GO:0043551	regulation of phosphatidylinositol 3-kinase activity	1	1	1.0	0.0284
